# Effect of 970 nm low-level laser therapy on orthodontic tooth movement during Class II intermaxillary elastics treatment: a RCT

**DOI:** 10.1038/s41598-021-02610-7

**Published:** 2021-12-01

**Authors:** Bénédicte Pérignon, Octave Nadile Bandiaky, Caroline Fromont-Colson, Stéphane Renaudin, Morgane Peré, Zahi Badran, Madline Cuny-Houchmand, Assem Soueidan

**Affiliations:** 1 Department of Orthodontics, UIC 11, CHU de Nantes, Nantes, France; 2 Division of Fixed Prosthodontics, University of Nantes, 44042 Nantes, France; 3 Biostatistics and Methodology Unit, Department of Clinical Research and Innovation, CHU Nantes, Nantes, France; 4 College of Dental Medicine, University of Sharjah, Sharjah, UAE; 5 Head and Chair of Periodontology Department, Faculty of Dental Surgery, UIC 11, Rmes U1229, University of Nantes, 1 Place Alexis Ricordeau, 44042 Nantes, France

**Keywords:** Lasers, LEDs and light sources, Clinical trial design, Randomized controlled trials

## Abstract

This prospective randomized clinical trial aimed to evaluate the effect of low-level laser therapy on tooth movement during Class II intermaxillary elastics treatment. Forty-two patients with Class II malocclusion were included, and their maxillary quadrants were allocated into two groups: treatment with an active diode laser and a placebo group. In each group, the time taken to obtain Class I occlusion after 6 months, rate of movement, total displacement of the maxillary canine to Class I occlusion and pain were recorded. The time to reach Class I occlusion in the active laser group (2.46 ± 2.1 months) was not significantly different from that in the placebo group (2.48 ± 2.0 months) (*p* = 0.938). Interestingly, the total distance of movement on the active laser side (2.27 ± 1.5 mm) was significantly greater than that on the placebo side (1.64 ± 1.3 mm) (*p* = 0.009). The pain levels on days 1, 2 and 3 were not significantly different between the laser and placebo sections. The rate of distance change toward Class I occlusion in the laser group (1.1 ± 0.7 mm/month) was significantly higher than that in the placebo group (0.74 ± 0.6 mm/month) (*p* = 0.037). Low-level laser therapy (970 nm) did not reduce the time needed to obtain Class I occlusion, but a significant acceleration in tooth movement was observed in the irradiated group.

Trial registration: NCT02181439. Registered 04 July 2014—https://www.clinicaltrials.gov/ct2/results?term=cinelaser.

## Introduction

The long duration of orthodontic treatments is often considered to present a risk of withdrawal from treatments or lead to a lack of patient cooperation. Long orthodontic treatments also result in an increased risk of root resorption, gingivitis, and tooth decay^1^. Thus, many studies have investigated various ways to accelerate orthodontic tooth movement, including the piezocision technique, corticotomy, photobiomodulation, low-level laser therapy, electric stimulation, pulsed electromagnetic fields, and mechanical and physical methods^2–5^.

Due to its biomodulatory action that stimulates the mechanisms of tissue remodeling, the low-level diode laser technique has been shown to be a promising noninvasive approach to accelerating tooth movement during orthodontic treatments^6–10^. Furthermore, diode lasers have been studied for their analgesic properties, especially when used during orthodontic treatments^11,12^. Confirmation of the ability of lasers to increase the rate of tooth movement may have a positive effect by reducing the duration of treatment and the risk of complications and increasing patient cooperation and motivation. The reduction of pain associated with laser therapy may also improve patient quality of life^13^.

Previous studies have been very heterogeneous regarding laser settings and irradiation protocols^14,15^. Moreover, previous studies have focused on the efficiency rather than on the clinical relevance of the protocol. In fact, previous studies have been conducted with lower wavelengths (from 670 to 904 nm)^16^, and treatments with higher wavelengths applied during low-level laser therapy (LLLT), which goes up to 980 nm, have not been well studied. However, recent studies on rats have shown encouraging results at these settings; furthermore, a wavelength of 970 allows deep penetration through soft tissue to reach alveolar bone and the periodontal ligament^17^.

The aim of this prospective randomized clinical trial was to evaluate the effect of low-level laser therapy on tooth movement during Class II intermaxillary elastics orthodontic treatment; in other words, we asked whether the low-level laser therapy (LLLT) side could reach Class I occlusion faster than the control side.

## Methods

This study was designed as a randomized controlled clinical trial with a split-mouth experimental design to determine the effect of low-level laser therapy on tooth movement during Class II intermaxillary elastics. The study followed the medical protocols and ethics described in the Declaration of Helsinki and was approved by the ethics committee of Nantes University Dental Hospital (IRB No. 2014-A00471-46). Both parents and patients were informed in detail about the possible risks and benefits, and all signed an informed consent form. The study data were collected in the orthodontic department of the University Hospital of Nantes between April 2015 and October 2017, and the trial protocol was registered at clinialtrials.gov (NCT02181439).

### Sample size calculation

The primary outcome was the time to obtain Class I occlusion after 6 months and was compared between the irradiated and nonirradiated areas (the patient was his own control). The clinically relevant difference to be demonstrated was set at 1 month. Assuming a standard deviation of the difference at 2 months and with an alpha risk set at 5% and a power at 80%, 34 patients were needed. To guarantee the necessary power, 20% more patients were included, i.e., 42 patients in total.

### Eligibility criteria

The inclusion criteria were healthy patients between 10 and 18 years of age who had a complete or partial bilateral Class II canine malocclusion, that is, any patient who presented a maxillary canine in a mesial position relative to the mandibular canine, had permanent teeth and required the use of intermaxillary forces for correction of dental Class II malocclusion. Patients who were receiving treatments that may interfere with orthodontic treatments or pain assessments were excluded from the study. Similarly, patients who had a one-sided, asymmetrical Class II malocclusion, had abnormalities of shape and/or structure involving the maxillary canines and the first mandibular molars resulting in an increased risk of bracket release, had number abnormalities, or were absent from at least three appointments were excluded from the study.

### Randomization

Randomization was employed using fixed blocks (ratio 1:1). The study statistician generated the allocation list using a secure computer-generated online remote system controlled by the independent research promotion unit at the University Hospital of Nantes, which had no role in patient recruitment. Maxillary quadrants of 42 patients were divided into two groups using the interactive web response system (IWRS): group A, with active laser treatment on the right side and inactive laser treatment on the left side, and group B, with the active laser on the left side and the inactive laser on the right side. Double-blinding was not possible due to the equipment used in this study. For each patient to have a proper control side, each patient received a laser application located either on the right maxillary quadrant or on the left maxillary quadrant. It should be noted that diode laser penetration is too low to affect the control side and exerts a localized action at the treated area. In addition, to facilitate data analysis, all irradiated sites were grouped into one group (laser A + B), and all nonirradiated sites were grouped into another group (placebo A + B).

### Clinical intervention

Orthodontic treatment was initiated for all patients by the bonding of bioprogressive Ricketts brackets manufactured by Rocky Mountain Orthodontics (RMO) and levelling and aligning of the maxillary and mandibular arches. Segmented blue 14’’ Elgiloy wires (0.016*0.022) (manufactured by RMO) were used in the maxilla, and a maximum cortical anchorage (45° coronolingual torque on the molar part of the utility arch also made of blue elgiloy) was applied at the mandibula for each participant. The segmented wires allowed an independence of movement between the left side and the right side^18^. Therefore, irradiation of one side could not influence the control side. Class II elastics were placed by the patient from the maxillary canine to the first mandibular molar to retract the maxillary canine. The elastic size was 5/6″, and the force level was 4½ OZ (manufactured by RMO). The distance between the maxillary canine tip and the mandibular canine tip was measured in the mouth with an electronic caliper during the first session and recorded for both sides before the first laser application. These measurements were performed by an operator trained in the use of the caliper to ensure reliability.

The laser used was a low-energy Sirolaser Advance (Sirona) diode laser with a 970 nm wavelength. Laser therapy was performed by a qualified and experienced operator (CF).

**Figure 1:**
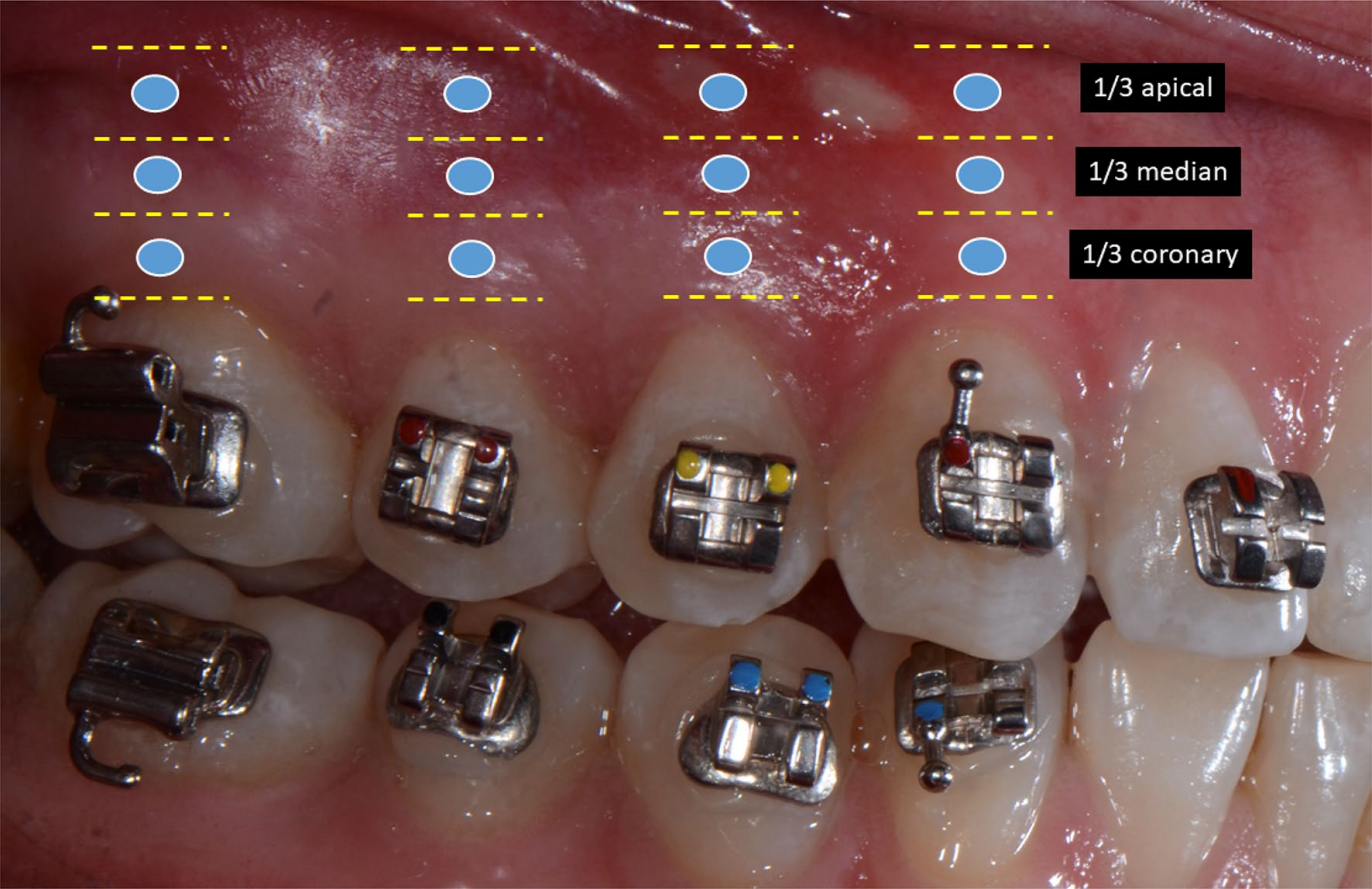
Vestibular laser application points.

After the consultation, during which the instructions for wearing the elastics were given to the patient, the patient was moved to a room dedicated to laser therapy. The operator randomized the maxillary quadrants to group A or group B. During this first session, the mucosal surfaces were air-dried, and the laser’s fiber tip was placed 5 cm from the mucosa to obtain an exposure diameter of 2 mm. The diode laser was activated in continuous mode (optical fiber 320 μm) for 2 s at a power of 0.5 Watts and with an energy of 30 J/Cm^2^. Thus, each exposure point received 0.9 J. Irradiation was performed at six points per tooth on four teeth (the canine, the first and second premolar, and the first maxillary molar). For each tooth, there were 3 vestibular application points and 3 palatal application points: one point at the coronary third of the root, one point at the median third and one point at the apical third (Fig. 1).

The total energy received by the irradiated side was 21.6 J. For the placebo side, the same procedure was applied with the exception that the laser was not activated and only the directional beam was enabled. Therefore, the same beeps at the start and end of the procedure were heard by the patient for both the irradiated side and the placebo side.

The second laser application session was carried out 1 month after the first application. The intercanine distances were measured again following the same protocol. Then, monthly monitoring and measurement visits were scheduled for up to 6 months after the first application to allow an independent investigator (BP) to evaluate the effectiveness of the laser treatment. To determine the coefficient assigned to the measured distance (− or +), the investigator noted the position of the mandibular canine forward or backward of the maxillary canine; a negative value corresponded to distal occlusion of the maxillary canine relative to the mandibular canine, and a positive value corresponded to the inverse situation. Thus, alignment of the maxillary and mandibular canine points corresponded to a value of 0.

### Outcome measure

The primary outcome of interest was the time taken to achieve Class I occlusion, which was compared between the experimental and placebo halves of the dentition. One month was considered a clinically significant difference, which corresponds to the minimum time between two appointments during orthodontic treatment. The secondary outcomes were total displacement of the maxillary canine to Class I occlusion, the pain level, and the speed to reach dental Class I occlusion.

**Figure 2:**
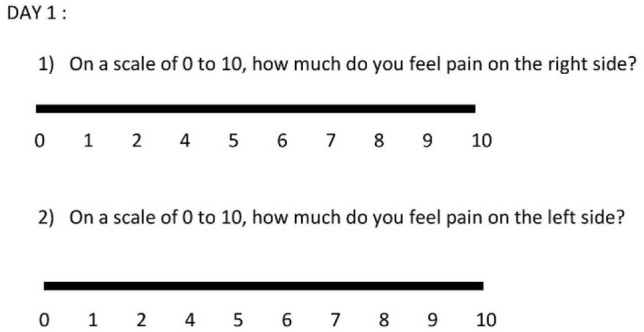
Example of the questionnaire measuring pain with a VAS.

The time to reach Class I occlusion was calculated as the difference between the first month (M1, M2, …, M6) in which Class I was observed during the clinical examination on both sides of the patient and the starting month (M0) of the treatment. The total displacement was calculated for each side by summing the displacements from each consult from M1 to M6. The displacement between two sessions was measured as the difference between the measured position determined in the previous month and that observed during the new appointment. Because the treatment is most painful during the first few days following placement of the elastics, the maxillary pain felt by the patient for each side was assessed during the first 3 days through a questionnaire that was filled out at home by the patient using a visual analog scale (VAS) (Fig. 2).

The speed of reaching Class I occlusion was calculated using two factors: the time required to reach Class I occlusion (the primary outcome) and the distances measured (the secondary outcome).

### Statistical analysis

eCRF Ennov Clinical software was used for data collection and tracking. The significance level was set at 5%. The sample size (n ≥ 30) allowed application of the central limit theorem, which states that if you have a population with the mean μ and the standard deviation σ and take sufficiently large random samples from the population with replacement, then the distribution of the sample means will be approximately normally distributed. The homogeneity of variance was only verified in tests between independent groups (with Levene’s test). A paired Student’s t-test was used for analysis of the data for the primary and secondary objectives. The descriptions include the numbers and percentages of modalities for the qualitative variables and the minimums, maximums, means, standard deviations and medians for the quantitative variables.

### Ethics approval and consent to participate

This study was approved by the ethics committee of (IRB No. 2014-A00471-46). Both parents and patients were informed in detail about the possible risks and benefits, and all signed an informed consent form.

## Results

### Participant flow

**Figure 3:**
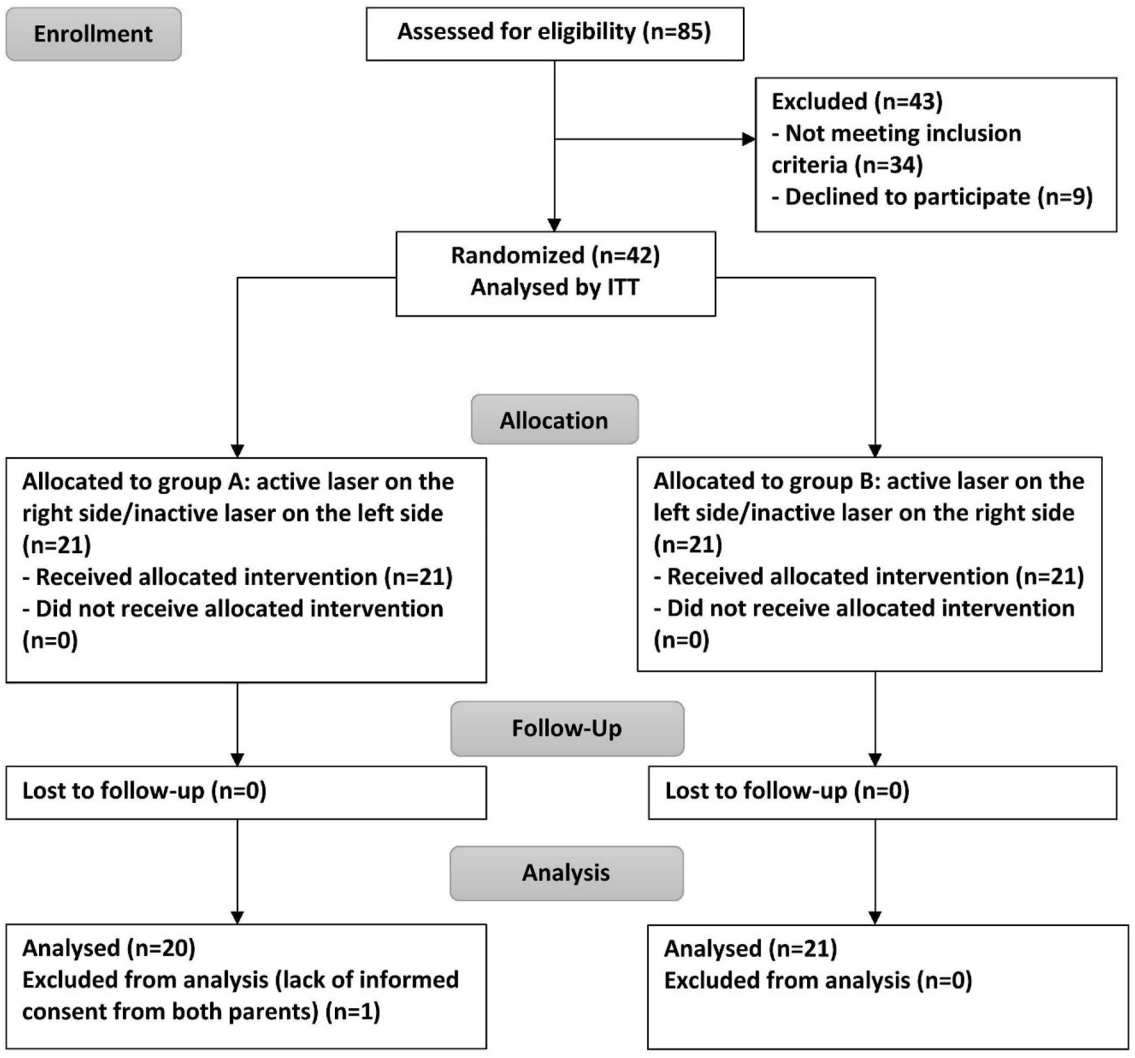
Flowchart of the study.

The study population consisted of 42 patients, who were randomized into two groups, A and B, according to a split-mouth experimental design. The characteristics of these patients are presented in Table 1. Only one patient was excluded from the analysis due to a lack of informed consent from one of the two parents (Fig. 3). Statistical analysis of the two situations (per person and intention to treat) did not show differences and led to the same conclusions. We have chosen not to show the results of the two situations to avoid making the text too wordy or dense and to facilitate interpretation of the results.

**Table 1:** Patient characteristics and mean distances between the maxillary and mandibular canine tips in the irradiated and placebo groups at baseline.

Variables	Group A	Group B	Total		
Age, Mean ± SD (Min–Max)	14 ± 1.4 (12–16)	13.1 ± 1.5 (10–16)	13.6 ± 1.5		
Men	5 (25%)	8 (38.1%)	13 (31.7%)		
Women	15 (75%)	13 (61.9%)	28 (68.3%)		
Distance between the maxillary and mandibular canine tips on the right Mean (SD)	− 1 (1.6)	− 1 (1.6)	− 1. (1.6)		
Distance between the maxillary and mandibular canine tips on the left Mean (SD)	− 1.6 (2)	− 1 (1.8)	− 1.2 (1.9)		
Class II confirmed on right/left, n (%)	20 (100%)	21 (100%)	41 (100%)		

	Groups	Mean (mm)	SD	95% CI	*p* value
Distance between maxillary and mandibular canine	Irradiated	− 1	1.7	− 1.5; − 0.4	0.0957
	Placebo	− 1.3	1.8	− 1.9; − 0.7	

*mm* millimeters, *SD* standard deviation, *95% CI* 95% confidence interval; significant at *p* < .05.

Therefore, the analysis was performed on 41 patients with bilateral Class II malocclusion: 13 males and 28 females, between 10 and 16 years of age.

Fourteen of the patients did not reach Class I occlusion on either side. However, these patient data were used for statistical analysis in intention-to-treat (ITT) for secondary objectives, which assessed the total displacement of the maxillary canine until Class I occlusion was reached and measured the speed at which the canines reached Class I occlusion on both sides.

Regarding the remaining secondary objective, which was to compare the pain levels on both sides of the maxilla using the VAS during the first 3 days of wearing Class II elastics, 32 patients were evaluated with the VAS at day 1 (D1) and D2. Nine of the patients did not complete the questionnaire on these dates. For the VAS at D3, only 31 people were evaluated because 10 patients did not complete the questionnaire by D3.

Despite randomization at the beginning of the study at D0, the mean distance between the maxillary and mandibular canine tips was statistically greater at a threshold of 9% on the irradiated side than on the placebo side, with a mean of − 1.3 ± 1.8 mm for the placebo half and − 1 ± 1.7 mm for the irradiated half (Table 1). Therefore, on average, the experimental side had a higher Class II malocclusion to correct than the placebo side.

### Main findings

Concerning the main objective, the time to reach Class I occlusion in the laser half was not significantly different from that in the placebo half. The laser sector had, on average, a Class I occlusion lead time of 2.46 months compared to 2.48 months (*p* = 0.93) in the placebo group (Table 2).

**Table 2:** Mean time to reach canine Class I occlusion in Class II elastics patients in the two groups.

	Groups	Mean (month)	SD	95% CI	*p* value
Time to reach Class I occlusion in patients wearing Class II elastics	Laser (n = 41)	2.46	2.1	1.8; 3.1	0.938
	Placebo (n = 41)	2.48	2.0	1.8; 3.1	

*SD* standard deviation, *95% CI* 95% confidence interval; significant at *p* < .05.

The required time to reach Class I was calculated by considering the entire population studied in the secondary objectives (concerning the distances and rates) to have better comparability between the results. Again, there was no significant difference between the irradiated and placebo halves (*p* = 0.764).

Regarding the results of the secondary objectives, the total displacement of the laser half was significantly greater than that of the placebo half (*p* = 0.009) (Table 3).

**Table 3:** Mean displacement of the maxillary canine to Class I in the two groups.

	Groups	Mean (mm)	SD	95% CI	*p* value
Total distance traveled by the maxillary canine to reach Class I	Laser (n = 27)	2.27	1.5	1.7; 2.8	0.009*
	Placebo (n = 27)	1.64	1.3	1.1; 2.2	

*mm* millimeters, *SD* standard deviation, *95% CI* 95% confidence interval; *Indicates significant differences between the groups.

For evaluation of pain, the mean differences in VAS scores from D0 to D1, D2 and D3 were not significantly different between the laser and placebo halves among the patients who completed the questionnaire (Table 4).

**Table 4:** Mean differences in evaluation of the VAS scores at D1, D2 and D3 in the two groups.

		Mean of differences between the laser and placebo halves	SD of differences between the laser and placebo halves	95% CI	*p *value
VAS at D1	32	− 0.2	2.5	− 1.1; 0.7	0.600
VAS at D2	32	0.1	2	− 0.6; 0.8	0.795
VAS at D3	31	0.2	1.8	− 0.4; 0.9	0.477

*VAS* visual analog scale, *D1* day 1, *D2* day 2, *D3* day 3, *SD* standard deviation; significant at *p* < .05.

Finally, the speed of reaching Class I occlusion (i.e., the rate of change toward Class I occlusion) in the laser half was significantly higher than that in the placebo half (*p* = 0.037) (Table 5).

**Table 5:** Mean rate to reach Class I occlusion in the two groups.

	Groups	Mean (mm/month)	SD	95% CI	*p* value
Rate to reach Class I (intention-to-treat population)	Laser (n = 27)	1.0	0.7	0.7; 1.3	0.037*
	Placebo (n = 27)	0.7	0.6	0.5; 1.0	

*mm* millimeters, *SD* standard deviation, *Indicates significant changes between the groups.

## Discussion

The aim of the study was to evaluate the efficiency of LLLT on the movement of teeth during the use of intermaxillary elastics to correct Class II malocclusion. We have chosen to limit the comparison of our results only to non-surgical and non-invasive techniques that are indicated in simple clinical situations. Invasive techniques used in quite different and complex indications have not been addressed in this work.

From the results of this study, it was not possible to conclude that laser irradiation at a 970 nm wavelength appreciably reduced the time to reach Class I occlusion. However, the results provide possible answers because the rate of displacement of the teeth was significantly higher in the irradiated half than in the placebo half. This result may be explained by the fact that, despite randomization of the patients, the distances on the irradiated side were greater than on the placebo side at the beginning of the trial. These results are similar to previous data reported in the literature. Indeed, Olyaee et al.^19^ reported in their systematic review that 8 out of 11 included studies showed that LLLT has a significant impact on acceleration of orthodontic tooth movement. Qamrubbin et al.^20^ obtained a canine retraction of 1.60 mm in 9 weeks on the irradiated side compared to a retraction of 0.79 mm on the placebo side. Similarly, Cruz et al.^8^ found a canine retraction of 4.39 mm at 60 days on the irradiated side compared to 3.0 mm on the placebo side (*p* < 0.001).

Confirmation of these results with a larger sample seems necessary, which would reduce the risk of a difference between the 2 groups on day 0. We chose to administer only two laser applications (at M0 and M1), unlike other studies in the literature. For example, Sousa et al.^21^ carried out an application at D1, D3 and D7 every month for 3 months. On the other hand, Genc et al.^22^ carried out irradiation at D0, D3, D7, D14, D21 and D28. However, these protocols require many appointments. Our irradiation schedule was chosen to closely mimic the actual conditions in clinical practice. In fact, seeing a patient several times in the same week to perform laser applications is challenging. The present study showed that despite the small number of applications, a higher rate of displacement was observed on the irradiated side. In future studies, it seems necessary to keep the protocol in line with clinical reality to evaluate the usefulness of such a device in current practice. In addition, studies^23,24^ on photobiomodulation or low-intensity pulsed ultrasound have also shown an acceleration of orthodontic tooth movement, but this required a daily application of light (10 min) or ultrasound (20 min) at home by the patient throughout the treatment period. These nonsurgical and noninvasive methods are more restrictive for the patient and require the purchase of equipment, thus increasing the cost of treatment.

In previous studies, some heterogeneity was observed in the laser parameters, including dose, fluence, wavelength and application time^25,26^. In the present study, the laser had a higher wavelength of 970 nm. In previous studies, the wavelength ranged from 670 nm for Dominguez et al.^27^, who reported a canine retraction of 3.73 mm on the laser side compared to 2.71 mm on the placebo side (*p* < 0.05), to 904 nm for Kansal^28^, who reported no significant difference between irradiated and nonirradiated sides. Considering the results of the present study, it can be supposed that a high wavelength may not interfere with the acceleration of dental movements, as the study by Kansal might suggest. However, the dose applied in our study was not higher (21.6 J) than doses used in previous studies. Indeed, according to the literature, it may be interesting to focus more on the dose parameter rather than on the wavelength or fluence. The dose may be the decisive factor in determining protocols that aim to establish an optimal therapeutic window^29,30^. Two studies that failed to show increased tooth movement applied a dose of 1.2 J and 108 J^28,31^. Thus, the ideal dose may be assumed to be within a wide range from 2 to 107 J. The studies that showed an increase in the rate of tooth movement with laser therapy applied a dose between 2 and 10 J^8,21^. Our study suggests possible acceleration of tooth movement with the use of a diode laser at a higher dose than applied in previous studies, but we used a dose that is still within the wide range.

Our method for measuring the distance could present a lack of accuracy and repeatability, especially with patients who have a blunt canine tip in which a constant mark is difficult to find. In future studies, a more precise mark might be chosen, for example, the distal or mesial side of the canine bracket. However, unless an indirect bonding protocol with individualized gutters on each tooth for precise repositioning of the bracket is used, potential release of the bracket may occur during the study, resulting in a loss of accuracy of the information collected after rebinding. Another solution would be to make digital prints at each appointment and to determine computer benchmarks to accurately calculate the displacements. This type of issue has not been found in the literature because all the studies on the acceleration of tooth movement were conducted in patients who had undergone extractions^8,21,31,32^. Thus, the measured variable was the space between the canine and the premolar on the same arch, which reduced imprecision of the measurement. However, unlike other studies that only looked for movement of a single tooth, our study seems to show significant results by showing movement of a whole segment (the canine, the first and second premolar and the molar), which suggests the possibility of using the laser not only for canine retraction but also for a wide range of clinical situations. Indeed, treatment of Class II malocclusion with the use of intermaxillary elastics is now widespread in orthodontics, and extractions are less common.

Regarding the pain questionnaire, establishing another pain evaluation after the second irradiation to remove the bias induced by arc change and the use of Class II elastics may yield interesting results. However, no significant difference in pain at the first application between sides was identified, but we cannot make conclusions based on this outcome because of the lack of power caused by an important number of non-answered questionnaires.

Additionally, asking the patient about the type, dose and number of analgesics taken after laser applications may have yielded interesting results. There may be different tooth movements depending on the medication used: ibuprofen, aspirin or paracetamol. Various studies have shown that the use of nonsteroidal anti-inflammatory drugs decreases tooth movement. Regarding ibuprofen, in 2004, Kleber et al.^33^ showed that the drug inhibited tooth movement in 26 rats under orthodontic forces of 50 and 100 g. Arias and Marquez-Orozco confirmed these results. They observed that 30 mg/kg ibuprofen led to a decrease in osteoclasts in the pressure area of the periodontal ligament and a significant decrease in orthodontic movements in rats in which the incisors received 35 g forces^34^. Under the same conditions, they also studied systemic administration of 100 mg/kg aspirin, where a decrease in osteoclasts and orthodontic movement was observed. In addition, in a study by Kleber, delivery of 17.5 or 35 mg/kg/day aspirin led to a decrease in molar displacement in rats under 50 or 100 g forces^33^. Paracetamol has no effect on orthodontic movement^35,36^. In a future study, it might be better if the patient only used paracetamol in the case of pain to avoid decreasing the speed of orthodontic movement.

When the protocol for the present study was written, it was decided that 42 patients should be included to increase the power of the study and claim a proper evidence level, which is 2 to 4 times higher than the average evidence level reported in previous studies (10–20 patients), and the study was designed as a randomized, controlled, double-blind study^37^. Despite this, some of the patients presented a major deviation from the protocol, that is, a lack of irradiation at M1 or three missing appointments. Notably, the selected population remained higher than the number of people included in other studies, even when analyzing results in a per protocol population (25 patients). Despite the large sample size and the use of randomization, a difference was observed between the intercanine distances on the placebo and laser sides at baseline. Therefore, in a future study, increasing the number of people included to obtain the same groups after randomization might be useful. In a future study, making the participants more aware of the study process to limit deviations from the protocol, such as absences for the second irradiation, unanswered questionnaires or missed appointments, could be interesting. However, these hazards are consistent with the real conditions for consultations in orthodontics at the CHU of Nantes. Our results must be interpreted with caution because their external validity remains limited. Indeed, due to the abovementioned limitations of our work and the fact that the study is monocentric and was carried out on a homogeneous population in a single nonrepresentative geographical area, generalization of the results of this study to the whole population is not possible. Other multicenter studies, different ethnic groups, and different appliance models should be studied.

## Conclusion

Based on our results and within the limitations of our sample size, we cannot conclude that the use of a diode laser reduces the time to obtain Class I status in patients wearing Class II elastics during orthodontic treatment. However, a significant impact on the acceleration of tooth movement was observed on the irradiated side.

## Data Availability

The datasets used and/or analyzed during the current study are available from the corresponding author on reasonable request.
